# Genome sequence and spore germination-associated transcriptome analysis of *Corynespora cassiicola* from cucumber

**DOI:** 10.1186/s12866-020-01873-w

**Published:** 2020-07-08

**Authors:** Shigang Gao, Rong Zeng, Lihui Xu, Zhiwei Song, Ping Gao, Fuming Dai

**Affiliations:** 1 Shanghai Runzhuang Agricultural Technology Co., Ltd, Shanghai, 201415 China; 2 Shanghai Engineering Research Centre of Low-carbon Agriculture, Institute of Eco-Environment and Plant Protection, Shanghai Academy of Agricultural Sciences, Shanghai, 201403 China

**Keywords:** *Corynespora cassiicola*, Cucumber, Genome sequence, Virulence-associated gene, Spore germination, RNA-Seq

## Abstract

**Background:**

*Corynespora cassiicola*, as a necrotrophic phytopathogenic ascomycetous fungus, can infect hundreds of species of plants and rarely causes human diseases. This pathogen infects cucumber species and causes cucumber target spot, which has recently caused large cucumber yield losses in China. Genome sequence and spore germination-associated transcriptome analysis will contribute to the understanding of the molecular mechanism of pathogenicity and spore germination of *C. cassiicola*.

**Results:**

First, we reported the draft genome sequences of the cucumber-sampled *C. cassiicola* isolate HGCC with high virulence. Although conspecific, HGCC exhibited distinct genome sequence differences from a rubber tree-sampled isolate (CCP) and a human-sampled isolate (UM591). The proportion of secreted proteins was 7.2% in HGCC. A total of 28.9% (4232) of HGCC genes, 29.5% (4298) of CCP genes and 28.6% (4214) of UM591 genes were highly homologous to experimentally proven virulence-associated genes, respectively, which were not significantly different (*P* = 0.866) from the average (29.7%) of 10 other phytopathogenic fungi. Thousands of putative virulence-associated genes in various pathways or families were identified in *C. cassiicola*. Second, a global view of the transcriptome of *C. cassiicola* spores during germination was evaluated using RNA sequencing (RNA-Seq). A total of 3288 differentially expressed genes (DEGs) were identified. The majority of KEGG-annotated DEGs were involved in metabolism, genetic information processing, cellular processes, the organismal system, human diseases and environmental information processing.

**Conclusions:**

These results facilitate the exploration of the molecular pathogenic mechanism of *C. cassiicola* in cucumbers and the understanding of molecular and cellular processes during spore germination.

## Background

Cucumber target spot caused by *C. cassiicola* (BerK & Curt) Wei has recently caused tremendous cucumber yield losses in China [1]. More importantly, *C. cassiicola*, as a necrotrophic parasitic fungus, can infect more than 500 plant species, including tomato, eggplant, tobacco, rubber, cotton, soybean and balsam pear, in addition to cucumber and cause plant spot diseases [2], which have transitioned from minor to major diseases in the last half century. Additionally, *C. cassiicola* is an opportunistic pathogen in humans and causes fungal keratitis under suitable conditions [3] and subcutaneous phaeohyphomycosis [4]. Therefore, the pathogenic mechanism of *C. cassiicola* against plants should be well studied to effectively control diseases caused by this pathogen.

For the past few years, research on the pathogenic mechanism of *C. cassiicola* has mainly focused on biological characteristics, pathogenicity differentiation, cloning of virulence-associated genes, etc. It was reported that *C. cassiicola* sampled from cucumber could grow at 10–35 °C, and its optimum growth temperature was approximately 30 °C [5]. *C. cassiicola* spores germinated from one end or both ends at 25–30 °C with > 90% relative humidity, and the germination rate in drops of water was the highest. *C. cassiicola* was able to invade cucumber leaves primarily through direct contact or via stomata [6]. *C. cassiicola* showed pathogenic and genetic variation among isolates sampled in different host plants, proving that the intraspecific strains of *C. cassiicola* showed high host specialization [2, 7]. Sixty-four *C. cassiicola* isolates from perilla, cucumber, tomato, aubergine and sweet pepper in Japan were divided into seven pathogenicity groups (PG1-PG7) [8]. Cassiicolin, a small secreted glycoprotein, is an important effector of *C. cassiicola*, and contains six different cassiicolin isoforms, *Cas1*, *Cas2*, *Cas3*, *Cas4*, *Cas5* and *Cas6*, in different *C. cassiicola* isolates sampled from various hosts and geographical origins [9–11]. The aggressive abilities of isolates were related to the type of isoform, and the isolates carrying the *Cas1* gene were the most aggressive to rubber trees. Additionally, some isolates with no *Cas* gene also generated moderate symptoms on rubber tree leaves, showing that uncharacterized effectors existed in *C. cassiicola* [10].

Similar to other filamentous fungal pathogens, *C. cassiicola* requires multiple pathogenic factors, such as cutinase, cell wall-degrading enzymes [12] and cytomembrane- and cell inclusion-degrading enzymes, in addition to the toxin (cassiicolin) to successfully invade host plants and cause disease, which are transported or regulated by multiple pathways of virulence-associated genes, including mitogen-activated protein kinase (MAPK), Ca^2+^ and cAMP signal pathways etc. [13]. To date, virulence-associated genes of *C. cassiicola* have rarely been cloned and functionally characterized except for two MAPK genes *CCk1* [14] and *CMP1* [15], and the cassiicolin-encoded gene *Cas* [16, 17], which are far from thoroughly representing the pathogenic mechanism of *C. cassiicola*. Thus, a great number of virulence-associated genes remain to be identified, cloned and functionally characterized.

The fungal conidium involved in reproduction is the main type of fungal asexual spore, and is the main form of inoculation and infection [18]. Resting conidia undergo germination and form sporeling hyphae or thalli under suitable conditions such as optimal humidity and temperature [19]. During this process, the progressive reduction in conidia hydrophobicity results in conidia swelling with isotropic growth and subsequent polarized growth, which is characterized as germ tube formation [20]. Conidium germination is usually essential for the fungal aggressiveness and colonization, which requires the involvement of a series of genes with different biochemical activities [18, 21, 22]. Therefore, spore germination-associated gene expression analysis will contribute to the understanding of the molecular mechanism of spore germination and pathogenicity, which has not been reported in *C. cassiicola* thus far.

Genome sequencing is a high throughput means to identify functional genes combined with homologous matches against functional databases or conserved domain searches [13, 23]. Furthermore, genome sequences accelerate the cloning of genes and their functional characterization, so it is imperative that the genome sequence of *C. cassiicola* be globally analyzed. Although the genome sequencing of two *C. cassiicola* isolates, CCP from a rubber tree and UM591 from the contact lens of a patient with keratomycosis, was completed [24, 25], it is still difficult to characterize the gene function in cucumber-sampled isolates due to high genetic variation among isolates from different hosts. RNA sequencing (RNA-Seq) is an effective technique for analyzing the expression of many genes and is widely used to identify differentially expressed genes (DEGs) between different treatments. Therefore, we first presented the draft genome sequence of a cucumber-sampled *C. cassiicola* isolate (HGCC) with high virulence to cucumber, and comparatively analyzed its genome with two other *C. cassiicola* isolates (CCP from a rubber tree and UM591 from a human) and other phytopathogenic ascomycete fungi in multiple families or pathways of virulence-associated genes in this study. Second, we studied the relative transcriptional levels of genes during the spore germination of *C. cassiicola* HGCC using RNA-Seq. These research results provide a great deal of information revealing the molecular mechanism of pathogenicity and spore germination of *C. cassiicola*.

## Results

### HGCC isolate features

**Fig. 1:**
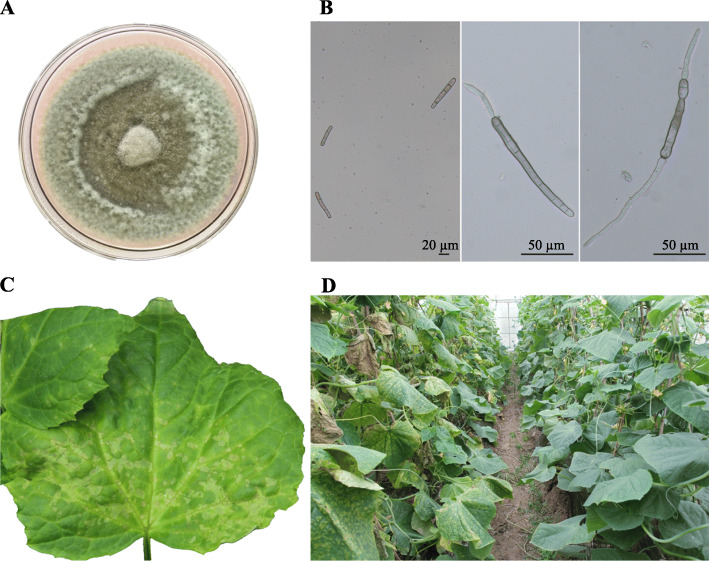
*C. cassiicola* isolate HGCC and cucumber target leaf spot symptoms. **a** HGCC mycelium colony on PDA medium 10 days after subculture at 25 °C in the dark. **b** Optical microscopy observation of HGCC conidia with/without germination in water. **c** Cucumber target leaf spot symptoms on a susceptible cucumber (Shenqing-1) leaf inoculated by HGCC with 1 × 10^5^ spores (60 h host inoculation). **d** Cucumber target leaf spot symptoms on the susceptible cucumber cultivar Shenqing-1 (left) and no obvious symptoms on the resistant cucumber cultivar Biyu (right) in a greenhouse in Shanghai

The HGCC isolate with high virulence to cucumber was selected for de novo sequencing of *C. cassiicola* sampled from cucumber. By being subcultured on potato dextrose agar (PDA) plates at 25 °C in the dark, HGCC produced a layer of fluffy aerial mycelium that was whitish gray when young and dark green when old (Fig. 1a) and easy to peel off. The clubbed conidia were varied in length (from 20 to 120 μm) and shape, had one to nine septa, and germinated at one or both ends in sterile distilled water (Fig. 1b). After artificial inoculation on sensitive and resistant cucumber cultivars, HGCC caused typical symptoms of cucumber target spot disease in the sensitive cultivar Biyu, but it hardly infected the resistant cultivar Shenqing-1 (Fig. 1c and d).

### Genome sequencing and general features

The HGCC genome was de novo sequenced (184 × Coverage) using an Illumina HiSeq X 10 system. A total of 54,580,316 high-quality reads were assembled into 1032 scaffolds (N50: 500 kb) with a genome size of 42.7 Mb, which was slightly less than that of CCP (44.8 Mb) (JGI: 1019537) and greater than that of UM591 (41.4 Mb) (GenBank: JAQF00000000.1) (Table 1). The HGCC genome encoded 14,631 genes through the coding sequence (CDS) prediction, which was close to the 14,560 of CCP and the 14,744 of UM591 (Table 1). The number of protein-encoding genes in *C. cassiicola* was significantly different (*P* = 0.011) from the average number (11,664) of 10 other phytopathogenic fungi, including *Curvularia lunata*, *Bipolaris maydis*, *Cercospora zeae-maydis*, *Parastagonospora nodorum*, *Setospaeria turcica*, *Pyrenophora tritici-repentis*, *Magnaporthe oryzae*, *Aspergillus flavus*, *Fusarium graminearum* and *Botrytis cinerea* (Additional file 1: Table S1). However, the number of secreted proteins in HGCC (1049) was less than that in CCP (1076) and UM591 (1106). The proportions of secreted proteins in the three isolates were 7.2% for HGCC, 7.4% for CCP and 7.5% for UM591, which were similar to those of the other phytopathogenic ascomycete fungi (7–10%) [23].

**Table 1:** Comparison of genome features among the three *C. cassiicola* isolates HGCC, CCP and UM591

Features	HGCC	CCP	UM591
Assembly size (Mb)	42.7	44.8	41.4
Scaffolds	1032	244	1941
GC (%)	51.78	51.89	52.47
Repeated sequences (%)	0.52	–	–
Protein-coding genes	14,631	14,560	14,744
Gene density (genes per Mb)	343	325	356
Average CDS length (Bp)	1575	1589	1539
Secreted proteins	1049	1076	1106

### Interspecific genome-wide phylogeny

**Fig. 2:**
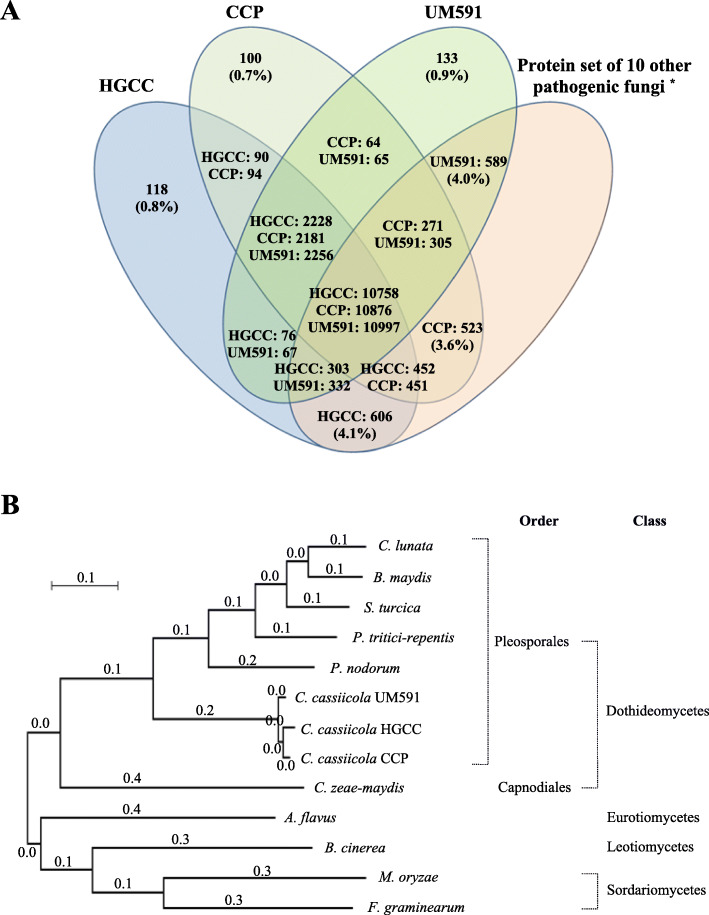
Comparative genomics and evolutionary analysis of *C. cassiicola*. **a** Reciprocal BLAST analysis of the protein sequences among three *C. cassiicola* isolates (HGCC, CCP and UM591) and other phytopathogenic fungi. ^*^ Protein set of 10 other phytopathogenic fungi: protein set of 10 other selected phytopathogenic fungi used in evolutionary analysis. **b** The evolutionary relationship of *C. cassiicola* with selected fungal species is shown by a maximum likelihood phylogenomic tree constructed using MEGA 7.0 software

The HGCC genome had a 91.6 and 90.5% amino acid sequence identity with CCP and UM591, respectively, and a 44.9–57.8% identity with other phytopathogenic fungi, such as *C. lunata* (57.4%), *B. maydis* (57.1%), *S. turcica* (57.0%), *P. nodorum* (57.8%), *P. tritici-repentis* (56.8%), *C. zeae-maydis* (47.0%), *A. flavus* (47.5%), *B. cinerea* (47.2%), *F. graminearum* (46.0%) and *M. oryzae* (44.9%). More than 70% of HGCC genes had a > 90.0% amino acid sequence identity with CCP (75.5%) and UM591 (72.4%), which was far more than the 55% between two *C. lunata* isolates, CX-3 from maize and m118 from sorghum [23]. A total of 12,986, 13,057 and 13,253 homologous core genes were screened by reciprocal blast analysis in HGCC, CCP and UM591, of which 2228, 2181 and 2256 were specific to 10 selected pathogenic fungi (Fig. 2a), respectively. Additionally, HGCC, CCP and UM591 had 4.9% (724), 4.3% (623) and 4.9% (722) specific genes compared to each other, of which 0.8% (118), 0.7% (100) and 0.9% (133) were specific to the 10 selected pathogenic fungi, respectively (Fig. 2a and Table 2). It was suggested that the three *C. cassiicola* isolates had distinct differences in their genomes, although HGCC had a high amino acid sequence identity with both CCP and UM591.

**Table 2:** Genome-wide analysis for *C. cassiicola* HGCC, CCP and UM591 gene sets

Characteristics	HGCC	CCP	UM591	core^**a**^	HG-CCP-UM591 core^**b**^	HG-CCP-UM591 specific^**c**^	HG specific^**d**^	CCP specific^**e**^	UM591 specific^**f**^	HGCC PHI
Protein-encoding gene	14,631	14,560	14,744	10,758	12,986	2228	118	100	133	4232
Secreted protein	1049	1076	1106	816	1003	187	3	1	7	358
PHI	4232	4298	4214	3484	4208	724	3	0	0	–
Protease	612	625	621	509	614	105	0	0	0	258
Glycoside hydrolase	262	262	265	223	262	39	0	0	0	141
Lipase	21	23	20	17	21	4	0	1	0	9
Glucanase	30	33	32	29	29	0	0	0	0	24
Glucosidase	13	13	16	10	10	0	0	0	0	13
Xylanase	7	9	9	6	6	0	0	0	0	4
Pectate lyase	34	33	33	28	34	6	0	0	0	30
Cutinase	8	8	9	7	8	1	0	0	0	5
Cellulase	14	17	16	14	14	0	0	0	0	5
Pectinesterase	4	4	5	4	4	0	0	0	0	4
MFS transporter	238	246	240	196	238	42	0	0	0	230
ABC transporter	51	50	48	41	51	10	0	0	0	51
P450	218	214	209	177	218	41	0	0	0	198
Protein kinase	154	156	151	127	154	27	0	0	0	150
Histidine kinase	11	12	11	9	11	2	0	0	0	11
GPCR	157	159	173	133	157	24	0	0	0	96
Pth11-like GPCR	62	61	57	50	62	12	0	0	0	53
CFEM-containing protein	18	19	19	19	20	1	0	0	0	12
Fungal specific transcription factor	202	217	212	162	202	40	0	0	1	187
Backbone gene for secondary metabolism	52	51	49	49	49	0	3	3	0	50

^a^ core: *C. cassiicola* HGCC, CCP and UM591 and 10 other phytopathogenic ascomycetes genes grouped by reciprocal BLAST analysis with a cutoff *E*-value of 1e-5; ^b^ HGCC-CCP-UM591 core: HGCC genes present in both CCP and UM591grouped by reciprocal BLAST analysis with a cutoff *E*-value of 1e-5; ^c^ HGCC-CCP-UM591 specific: HGCC genes present in both CCP and UM591 but specific compared to 10 other fungi grouped by reciprocal BLAST analysis with a cutoff *E*-value of 1e-5; ^d^ HG specific: specific genes of HGCC against CCP, UM591, and 10 other fungi; ^e^ CCP specific: specific genes of CCP against HGCC, UM591, and 10 other fungi; ^f^ UM591 specific: specific genes of UM591 against HGCC, CCP, and 10 other fungi

A total of 11,111 gene-encoding proteins were classified into 3892 conserved protein families in HGCC by Pfam matches with profile hidden Markov models, which was close to the 3847 families containing 11,187 proteins in CCP and the 3860 families containing 11,110 proteins in UM591. Glycoside hydrolase (GH), pectate lyase, cutinase and cellulose were important pathogenic factors that were highly abundant in *C. cassiicola* (Additional file 1: Table S2). There was significant difference in the numbers of transporters (*P* = 0.043), the major facilitator superfamily (MFS) (*P* = 0.043), cytochrome P450 enzymes (CYPs) (*P* = 0.011), G protein-coupled receptors (GPCRs) (*P* = 0.028), Pth11-like G protein-coupled receptors (*P* = 0.011), protein kinases (*P* = 0.011), proteases (*P* = 0.011), cysteine proteases (*P* = 0.017), metallo proteases (*P* = 0.022), serine proteases (*P* = 0.011), GHs (*P* = 0.011), pectate lyases (*P* = 0.011), pectinesterases (*P* = 0.044), cellulases (*P* = 0.045), glucosidases (*P* = 0.010) and secondary metabolite backbone genes (*P* = 0.042) between *C. cassiicola* and the 10 other phytopathogenic fungi, and the number of proteins in the each family in the former was more than that in the latter, suggesting that *C. cassiicola* had family expansions in these families. These protein families were expected to play important roles in fungal survival in various adverse environments.

To mine potential virulence-associated genes, BLASTP searches of genomes of the three *C. cassiicola* and other phytopathogenic fungi were conducted against the pathogen-host interaction (PHI) database at a *E*-value of 1e-5. A total of 28.9% (4232) of HGCC genes, 29.5% (4298) of CCP genes and 28.6% (4214) of UM591 genes were matched with PHI database, respectively. There was no significant difference (*P* = 0.866) in the percentage of PHI-associated genes between the three *C. cassiicola* and the 10 other phytopathogenic fungi (average: 29.7%) (Additional file 1: Table S1).

An interspecific phylogenomic tree of *C. cassiicola* and the 10 other phytopathogenic fungi was constructed based on concatenated amino acid sequences of 2831 orthologous proteins (Fig. 2b). The tree showed that *C. cassiicola* of the Pleosporales order had genetic affinity with other fungi of the Pleosporales order, followed by *C. zeae-maydis* of the Capnodiales order of the Dothideomycetes class, *A. flavus* of the Eurotiomycetes class, *B. cinerea* of the Leotiomycetes class, and *M. oryzae* and *F. graminearum* of the Sordariomycetes class. In addition, it was shown in the tree that *C. cassiicola* speciation had occurred before the speciation of the other Pleosporales fungi, suggesting that *C. cassiicola* speciation had occurred before speciation of the Pleosporales order. These results were similar to the results of David Lopez [24].

### Pathogenic signal pathway

GPCRs transduce external environmental signals by way of heterotrimeric G proteins into secondary messengers to regulate gene expression and the subsequent cellular response [26]. These proteins are required in plant recognition and pheromone/nutrient sensing of phytopathogenic fungi [27]. Pth11, as one GPCR of *Magnaporthe grisea*, mediates appressorium differentiation and fungal pathogenicity [28], and its homologues exist in other phytopathogenic fungi. The HGCC genome contained 157 GPCRs, which was close to the 159 in CCP, less than the 173 in UM591. The number of GPCRs in *C. cassiicola* was significantly different (*P* = 0.028) from the average (113) of the 10 other phytopathogenic fungi (Additional file 1: Table S3). These GPCRs of *C. cassiicola* were classified into 9 classes, including A, B, C, D, E, F, MPR, PTH11 and STM1. The numbers of GPCRs of A (67 in HGCC, 68 in CCP and 87 in UM591), C (7 in HGCC, 8 in CCP and 11 in UM591) and PTH11 (62 in HGCC, 61 in CCP and 57 in UM591) classes in *C. cassiicola* were significantly different (*P* < 0.05) from the average (50 in A class, 5 in C class and 37 in PTH11 class) of the other phytopathogenic fungi, respectively. More than 80% of Pth11-like GPCRs were identified as PHI-associated genes in HGCC (53/62), CCP (50/61), UM591 (48/57)), and the 10 other phytopathogenic fungi (average: 32/37), respectively. The G protein alpha subunit is an important component of the heterotrimeric G protein complex [29] and can activate downstream effectors and plays an important role in fungal pathogenicity [30, 31]. In the HGCC genome, three G protein alpha subunits were identified, all of which were PHI-associated genes and showed high amino acid identities with a G protein beta subunit of *Pseudocercospora fijiensis* (GenBank: XP_007925889.1, 93%) and two G protein alpha subunits of *Stemphylium lycopersici* (GenBank: KNG46771.1, 95%; GenBank: KNG45504.1, 85%), respectively.

**Fig. 3:**
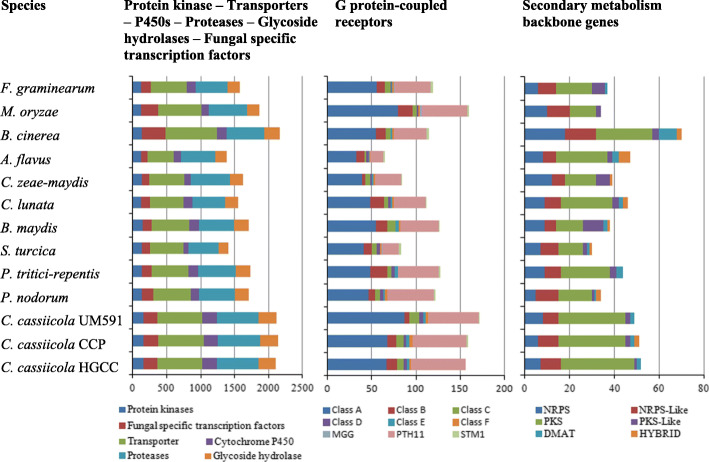
Virulence-associated protein family analysis of *C. cassiicola* and other fungi. NRPS, non-ribosomal peptide synthetase; PKS, polyketide synthetase; DMAT, dimethylallyl tryptophan synthase; HYBRID, hybrid PKS-NRPS enzyme

MAPK, cAMP and Ca^2+^ signaling are part of major signaling pathways and are associated with virulence in phytopathogenic fungi, which are controlled by a series of protein kinases [13]. Three MAPK pathways of *Saccharomyces cerevisiae*, FUS3/KSS1, Mpk1 and Hog1, have been well studied and are highly conserved in other fungi [13]. Based on homologous searches of the HGCC genome against known MAPK, cAMP or Ca^2+^ pathway-associated genes in *S. cerevisiae*, 48, 12 and 23 screened genes in HGCC were highly homologous with MAPK, cAMP and Ca^2+^ signal pathway genes of *S. cerevisiae*, respectively (Additional file 1: Tables S4, S5 and S6). A total of 154, 156 and 151 protein kinases were identified in HGCC, CCP and UM591, respectively, which were significantly different (*P* = 0.011) from 116 to 140 in other phytopathogenic fungi (Fig. 3, Additional file 1: Table S7). These protein kinases were classified into 8 classes. There was significant difference (*P* < 0.05) in the numbers of protein kinases in the Other and STE classes between *C. cassiicola* and the other phytopathogenic fungi, respectively. The STE class (16 kinases for HGCC, 15 for CCP, and 17 for UM591) and MAPK family (4 kinases for HGCC, 3 for both CCP and UM591) in the CMGC class were involved in the MAPK pathway. The PKA family (3 kinases for HGCC, 2 for both CCP and Um591) of the AGC class was involved in the cAMP pathway. The CLK family (1 kinase for HGCC, 3 for CCP, and 2 for UM591) and the RCK family (1 kinase for HGCC, 2 for CCP, and 1 for UM591) of the CMGC class, the CAMK class (20 kinases for HGCC, 18 for CCP, and 20 for UM591), and the PKC family (1 kinase for each of HGCC, CCP and UM591) in the AGC class were related to the Ca^2+^ pathway. Interestingly, almost all protein kinases were PHI-associated genes in HGCC (150/154), CCP (154/156), UM591 (148/151), and the 10 other phytopathogenic fungi (average: 125/129), suggesting that protein kinases played key roles in pathogenic processes mainly mediated by the three pathogenic signal pathways.

Histidine kinase (HK) phosphorelay signaling is usually used by fungi, bacteria, plants and slime molds to sense and adapt to their environment [32]. Fungal HK phosphorelay signaling mediates multiple biological processes, including virulence, differentiation, stress response and the biosynthesis of secondary metabolites [32, 33]. The HK signaling pathway is also known as a two-component signaling pathway containing a sensor HK and a response regulator (RR). HGCC, CCP and UM591 contained 11, 12 and 11 HKs, respectively, which were not significantly different (*P* = 0.492) from the average (10) of the other phytopathogenic fungi (Additional file 1: Table S7). It is worth noting that all HKs were PHI-associated genes.

### Protein families involved in degrading the plant cuticle, cell wall, cytomembrane and cell inclusion

To successfully invade the host plant, phytopathogenic fungi are expected to produce and secrete multiple extracellular degrading enzymes, such as cutinase for the degradation of the cuticle [34], cell wall-degrading enzymes (pectinase, cellulase and hemicellulase) [35], and cytomembrane- and cell inclusion-degrading enzymes (protease and lipase) [36, 37]. Eight, 8 and 9 cutinases were identified in the HGCC, CCP and UM591 genomes, respectively (Additional file 1: Table S2), which were not significantly different (*P* = 0.051) from the average (11) in the 10 other phytopathogenic fungi. There was significant difference (*P* = 0.011) in the number of pectate lyases, a type of pectinase, between *C. cassiicola* (34 in HGCC, 33 in CCP, and 33 in UM591) and the other phytopathogenic fungi (average: 12). However, pectinesterase, another type of pectinase, was less abundant than pectate lyase in *C. cassiicola* (4 in HGCC, 4 in CCP, and 5 in UM591) and the other phytopathogenic fungi (0–5). Fourteen, 17 and 16 cellulases were identified in HGCC, CCP and UM591, respectively, which were significantly different (*P* = 0.045) from the average (12) in the other phytopathogenic fungi. The hemicellulase glucanase (30 in HGCC, 33 in CCP, and 32 in UM591), glucosidase (13 in HGCC, 13 in CCP, and 16 in UM591) and xylanase (7 in HGCC, 9 in CCP, and 9 in UM591) were abundant in *C. cassiicola*, of which only glucosidase showed significant difference (*P* = 0.010) in number between *C. cassiicola* and the other phytopathogenic fungi (average: 9). More than half of cutinases and xylanases and almost all of pectate lyases, pectinesterases, glucanases and glucosidases were PHI-associated genes in these phytopathogenic fungi.

Proteases were very abundant in the *C. cassiicola* genomes (612 in HGCC, 625 in CCP, and 621 in UM591), which were significantly different (*P* = 0.011) from the average value (521) of the other phytopathogenic fungi (Additional file 1: Table S8). More than 40% of proteases were PHI-associated genes in HGCC (258/612), CCP (261/625), UM591 (254/621), and the 10 other phytopathogenic fungi (average: 226/521), respectively. These proteases were classified into 6 superfamilies, including the serine protease, metallo peptidase, cysteine peptidase, aspartic peptidase, threonine peptidase and glutamic peptidase superfamilies, the first three of which were the largest. The numbers of the serine proteases (355 in HGCC, 364 in CCP and 370 in UM591), metallo peptidases (136 in HGCC, 141 in CCP and 133 in UM591) and cysteine peptidases (74 in HGCC, 73 in both CCP and UM591) in *C. cassiicola* were significantly different (*P* < 0.05) from the average of the other phytopathogenic fungi (276 serine proteases, 111 metallo peptidases, and 65 cysteine peptidases) (Fig. 3, Additional file 1: Table S8). Aspartic peptidases are virulence factors in both plant and mammalian pathogens due to their ability to cleave a large number of host proteins [38]. *C. cassiicola* contained 11, 10 and 9 A11 transposon peptidases of the aspartic peptidase superfamily in HGCC, CCP and UM591, respectively. HGCC, CCP and UM591 contained 21, 23 and 20 lipases, respectively, which were not significantly different (*P* = 0.864) from the average (20) of the other phytopathogenic fungi (Additional file 1: Table S2).

GH catalyzes the hydrolysis of glycosidic bonds in complex sugars [39], playing a role in fungal pathogenesis. *C. cassiicola* contained 44 GH families (Additional file 1: Table S9). There were 262 GHs in HGCC, 262 in CCP, and 265 in UM591, which were significantly different (*P* = 0.011) from the average (194) in the other phytopathogenic fungi. Many GH families exhibited gene expansion in *C. cassiicola* when compared to the other phytopathogenic fungi, including the GH1, GH2, GH3, GH7, GH12, GH20, GH27, GH35, GH43, GH53, GH61, GH72, GH88, GH106 and GH107 families; however, the GH30 and GH 65 families exhibited gene constriction. More than half of GHs were PHI-associated genes in HGCC (141/262), CCP (138/262), UM591 (141/265), and the other phytopathogenic fungi (average: 100/194). Interestingly, almost all GHs of the GH3, GH7, GH10, GH17, GH18, GH20, GH28, GH31, GH61 and GH72 families were PHI-associated genes in *C. cassiicola* and the 10 other phytopathogenic fungi. It was reported that the GH6, GH7, GH45 and GH61 cellulases and GH10 xlyanases were absent in insect pathogenic fungi but present in phytopathogenic fungi [40]. The GH families of cellulases were abundant in HGCC (69), CCP (70) and UM591 (70), including GH3 (25 in HGCC, 26 in CCP, and 26 in UM591), GH 6 (2 each in HGCC, CCP, and UM591), GH7 (6 each in HGCC, CCP, and UM591), GH45 (2 each in HGCC, CCP and UM591), and GH61 (34 each in HGCC, CCP and UM591) cellulases. The GH16 family of xyloglucosy transferases plays an important role in the digestion of plant cell walls, and was abundant in HGCC (15), CCP (15) and UM591 (16) at values close or equal to the average (15) of the other phytopathogenic fungi.

### Protein families for transportation

*C. cassiicola* contained a large number of transporters (664 in HGCC, 669 in CCP, and 665 in UM591), which were significantly different (*P* = 0.043) from the average (548) of the other phytopathogenic fungi (Additional file 1: Table S10). More than 70% of transporters were PHI-associated genes in *C. cassiicola* HGCC, CCP, UM591 and the other phytopathogenic fungi. The MFS (238 in HGCC, 246 in CCP and 240 in UM591) and ATP-binding cassette (ABC) superfamily (51 in HGCC, 50 in CCP and 48 in UM591) superfamily were the two largest superfamilies of transporters. The former is capable of transporting small solutes in response to chemiosmotic ion gradients, and the latter transports small molecules and macromolecules under ATP hydrolysis [41, 42]. There was significant difference (*P* = 0.043) in the number of MFS transporters between *C. cassiicola* and the other phytopathogenic fungi (average: 160). However, there was no significant difference (*P* = 0.127) in the number of ABC transporters between *C. cassiicola* and the other phytopathogenic fungi (average: 43). Notably, almost all MFS transporters and all ABC transporters were PHI-associated genes in these phytopathogenic fungi, showing that MFS and ABC transporters played key roles in fungal pathogenicity.

In phytopathogenic fungi, drug transporters of ABC and MFS superfamilies can secrete endogenous fungal virulence factors such as toxins and protect pathogens against exogenous plant defense compounds such as phytoalexins [40, 43]. Two drug: H^+^ antiporter (DHA) subfamilies (DHA1 and DHA2), drug transporters of the MFS superfamily, can secrete toxic compounds into the outer environment [44]. The multidrug resistance (MDR) and pleiotropic drug resistance (PDR) subfamilies are drug transporters of the ABC superfamily that are capable of functioning in antifungal agent resistance [44]. There was almost no difference among the HGCC, CCP and UM591 genomes in the numbers of DHA1 (42, 43 and 44, respectively), DHA2 (5, 7 and 7, respectively), MDR (10, 9 and 9, respectively) and PDR (15, 15 and 14, respectively) (Additional file 1: Table S11), but there was significant difference (*P* = 0.042) in the number of DHA1 transporters between *C. cassiicola* and the other phytopathogenic fungi (average: 32). Interestingly, all drug transporters were PHI-associated genes except for DHA2 (4/5).

### Protein families for detoxification

CYPs are proteins of a superfamily, are ubiquitous in all biological kingdoms, and contain heme as a cofactor and are therefore hemoproteins. Fungal CYPs play key roles in various metabolic processes, such as housekeeping biochemical reactions, detoxification of chemicals and adaptation to adverse surroundings [45]. A large number of CYPs were identified in the *C. cassiicola* HGCC (218), CCP (214) and UM591 (209) genomes and classified into 112 families; there was significant difference (*P* = 0.011) in the number of CYPs between *C. cassiicola* and the other phytopathogenic fungi (average: 122) (Fig. 3, Additional file 1: Table S12). Interestingly, more than 90% of CYPs were involved in PHI in HGCC (198/218), CCP (196/214), UM591 (194/209) and the other phytopathogenic fungi (average: 111/122). The CYP65 and CYP505 subfamilies participate in the biosynthesis of mycotoxins; for example, the CYP65 subfamily catalyzes the epoxidation reaction in trichothecene biosynthesis in *F. graminearum* [46], and both CYP65 and CYP505 are required in fumonisin biosynthesis by *Fusarium verticillioides* [47, 48], showing that the CYP65 and CYP505 subfamilies were probably related to mycotoxin biosynthesis in *C. cassiicola*. CYP65 was the largest subfamily in the CYP superfamily. There was significant difference (*P* = 0.011) in the number of CYP65s between *C. cassiicola* (22 in HGCC, 23 in CCP and 25 in UM591) and the other phytopathogenic fungi (average: 11). Additionally, CYP505 was scarce in *C. cassiicola* (5 in HGCC, 4 in CCP and 5 in UM591) and the other phytopathogenic fungi (average: 3).

### Secondary metabolite backbone genes

Melanin and mycotoxin are important virulence factors in phytopathogenic fungi [49]. To data, melanin- and mycotoxin-associated genes have not yet been identified in *C. cassiicola* except for the cassiicolin-coded *Cas* gene. Secondary metabolite backbone genes are essential for the biosynthesis of melanin and mycotoxin as secondary metabolites. There was significant difference (*P* = 0.042) in the number of backbone genes between *C. cassiicola* (52 in HGCC, 51 in CCP and 49 in UM591) and the other phytopathogenic fungi (average: 42) (Additional file 1: Table S13). Notably, almost all backbone genes (50/52) were PHI-associated genes, showing that they were probably involved in the pathogenic process of these phytopathogenic fungi. The backbone genes of HGCC and UM591 were classified into 5 groups, including non-ribosomal peptide synthetase (NRPS), NRPS-like, polyketone synthase (PKS), PKS-like, dimethylallyl tryptophan synthase (DMAT), excluding hybrid PKS-NRPS enzyme (HYBRID), but which existed in CCP. PKS was the largest group in phytopathogenic fungi. The numbers of PKSs in *C. cassiicola* (33 in HGCC, 30 in CCP, 30 in UM591) were significantly different (*P* = 0.011) from that of the other phytopathogenic fungi (average: 17).

**Fig. 4:**
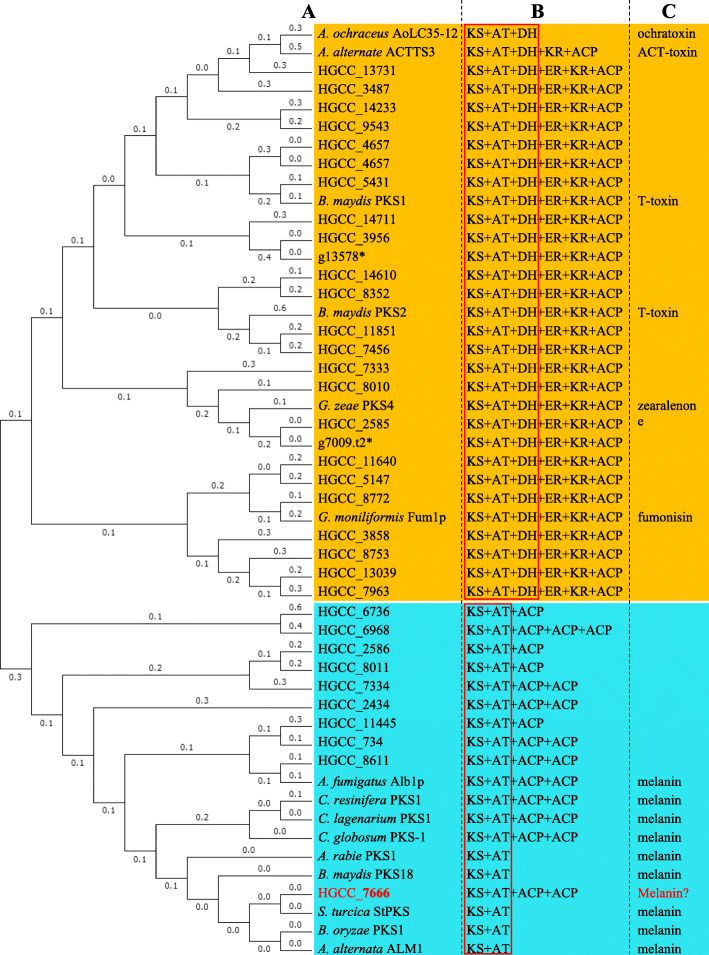
Phylogenetic and domain analysis of polyketide synthases (PKS) in *C. cassiicola* HGCC and other fungi. **a** A maximum likelihood tree of ketoacyl CoA synthase (KS) domain sequences of PKS in selected fungi. **b** Domain analysis of these PKSs using the SBSPKS database. Domain definitions were as follows: KS, ketoacyl CoA synthase; AT, acyltransferase domain; DH, dehydratase domain; ER, enoyl reductase domain; KR, ketoreductase domain; ACP, acyl carrier protein domain. **c** Toxins and melanin related to reported PKS in other fungi, the accession No. of which are shown in the “Methods” section. ^*^g13578 and ^*^g7009 are specific in CCP when compared to HGCC

To analyze the relationship between the PKS domain and its function, phylogenetic analysis for the ketoacyl CoA synthase (KS) domain of PKS was performed among *C. cassiicola* PKSs and role-known PKSs in other pathogenic fungi (Fig. 4). These PKSs were divided into two different clusters based on the phylogenetic analysis. One cluster was reducing PKSs with KS, acyltransferase (AT) and dehydratase (DH) domains at a minimum, which contained 25 *C. cassiicola* PKSs and 6 known PKSs involved in the biosynthesis of mycotoxins in the other phytopathogenic fungi, such as *Aspergillus ochraceus* AoLC35–12 for ochratoxin production, *Alternaria alternate* ACTTS3 for ACT-toxin production, *B. maydis* PKS1 and PKS2 for T-toxin production, *Gibberella zeae* PKS4 for zearalenone production, and *Gibberella moniliformis* Fum1p for fumonisin production. Two reducing PKSs (g13578 and g7009) were specific to CCP when compared to HGCC. The second cluster contained nonreducing PKSs with KS and AT domains at a minimum and did not include dehydratase (DH), enoyl reductase (ER) and ketoreductase (KR) domains; this cluster contained 10 PKSs of *C. cassiicola* and 9 known PKSs related to melanin biosynthesis in other fungal pathogens such as *Aspergillus fumigatus* Alb1p, *Ceratocystis resinifera* PKS1, *Colletotrichum lagenarium* PKS1, *Chaetomium globosum* PKS-1, *Ascochyta rabie* PKS1, *B. maydis* PKS18, *S. turcica* StPKS, *Bipolaris oryzae* PKS1, and *A. alternata* ALM1. It was difficult to identify which reducing PKSs were involved in mycotoxin biosynthesis in *C. cassiicola* due to the complicated evolutionary relationship for the KS domain of the reducing PKSs in *C. cassiicola* and known mycotoxin-related PKSs in other fungi. Nevertheless, the nonreducing PKS HGCC_7666 of *C. cassiicola* had the closest evolutionary relationship with known melanin-associated nonreducing PKSs, suggesting that HGCC_7666 was probably the backbone gene for melanin synthesis in *C. cassiicola*.

### Small, cysteine-rich peptides and effector proteins

Small cysteine-rich proteins (SCRPs) can be secreted directly into host plant cells to function in host recognition or colonization [50] and the stimulation of the host hypersensitive response (HR) [51]. Some SCRPs, as virulence effectors, facilitate fungal virulence in multiple ways, including host cell signaling perturbation, interference with pathogen recognition by the host and suppression of pathogen-associated molecular pattern (PAMP)-triggered immunity (PTI) [52]. Some SCRPs, such as *Avr* genes, trigger or suppress effector-triggered immunity (ETI) mediated by a gene-for-gene system in PHI [53]. Fifty-nine SCRPs were identified in HGCC ranging in size from 69 to 150 amino acids, including 1 hydrophobin, 1 cerato-platanin, 1 CFEM and 3 *Avr*-encoded proteins (AvrLm4–7) that were putative effector proteins (Additional file 1: Table S14). Fungal hydrophobins are involved in surface recognition [54], and play a role as effectors in the PHI [55]. Cerato-platanin family proteins could elicit disease resistance responses in the host plant [56].

Lysin motif (LysM)-containing effectors are ubiquitous in phytopathogenic fungi and are secreted out of the cell to suppress the immune response of the host [57, 58]. Common in the fungal extracellular membrane (CFEM) domain is a fungi-specific domain containing eight cysteines that is found in some proteins with proposed roles in fungal infection and colonization [59, 60]. HGCC contained 6 LysM-containing and 18 CEEM-containing genes that were candidate effectors and were probably involved in the fungal pathogenic process.

### Gene expression in spore germination

**Fig. 5:**
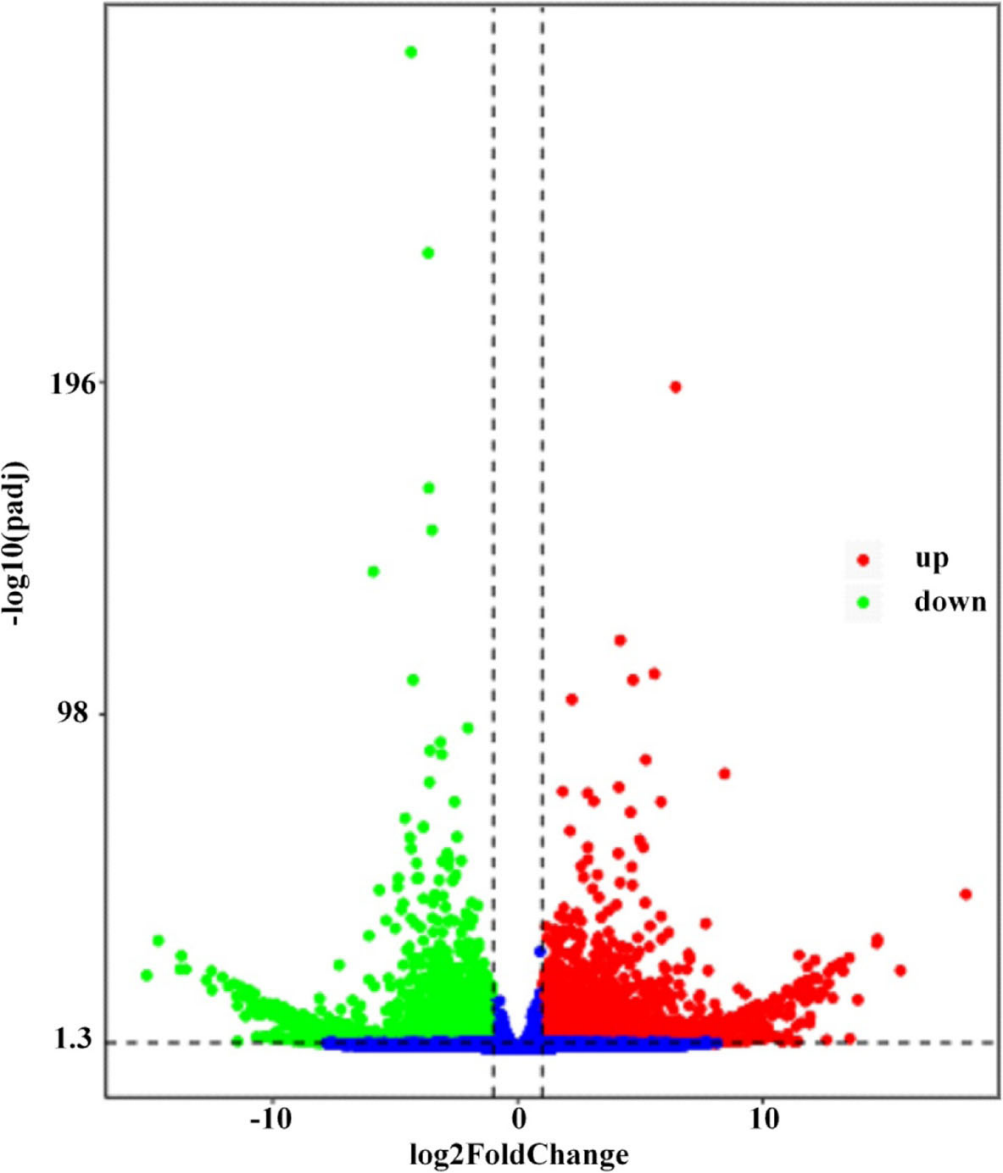
Volcano plot for global comparison of transcript profiles between germinated and ungerminated spores. Red points indicate up-regulated genes with a threshold of log2FoldChange > =1 and padj < 0.05. Green points indicate down-regulated genes with a threshold of log2FoldChange < = − 1 and padj < 0.05

To reveal the molecular and cellular processes during spore germination of *C. cassiicola*, 6 h- and 12 h-germination times were selected for RNA-Seq based transcriptome analysis. After 12 h at 25 °C in sterile ddH_2_O, the majority of spores germinated at two ends, and only a few germinated at one end (Fig. 1B). Biological triplicate samples of germinated and ungerminated spores of *C. cassiicola* were harvested for RNA isolation. RNA-Seq libraries were constructed and sequenced using Illumina HiSeq X ten. Following quality control and adapter trimming, 611,310,902 bp clean paired reads were obtained from 6 *C. cassiicola* RNA-Seq libraries. The sequencing yield from individual libraries ranged from 84,671,942 to 116,800,420 reads per sample (Table S15 in Additional file 2). Pearson correlation analysis showed that samples of the same treatment were highly correlated with each other in gene expression levels, with the R^2^ values of 0.885–1, which were higher than the R^2^ values of 0.742–0.85 between samples of different treatments (Fig. S1), suggesting that samples in the same treatment group were repeatable and that the RNA-Seq data were reliable. A total of 66–73% of *C. cassiicola* filtered reads per library mapped to the gene predictions of *C. cassiicola*. A total of 3288 genes were differentially expressed, of which 1552 and 1736 were up-regulated and down-regulated, respectively, during spore germination of *C. cassiicola* with ungerminated spores as the reference (Fig. 5). Specific DEGs are listed in Table S16 in Additional file 2. These DEGs contained hundreds of previously identified functional genes from the genome sequences classified by gene families (Table 3).

**Table 3:** Spore germination-related gene families

Gene family	Gene number	Up-regulated gene number	Down-regulated gene number
GPCR	63	18	45
G protein alpha subunit	1	1	0
Protein kinase	36	19	17
Glycoside hydrolase	76	42	34
P450	102	23	79
Protease	234	110	124
Pectate lyase	13	4	9
Cellulase	8	4	4
Cutinase	4	1	3
Glucanase	12	8	4
Glucosidase	4	4	0
Xylanase	2	1	1
SCRP	18	5	13
PKS	12	1	11
NRPS	3	0	3
NRPS_like	2	1	1
Transporter	243	94	149
ABC transporter	15	3	12
MFS transporter	94	33	61

**Fig. 6:**
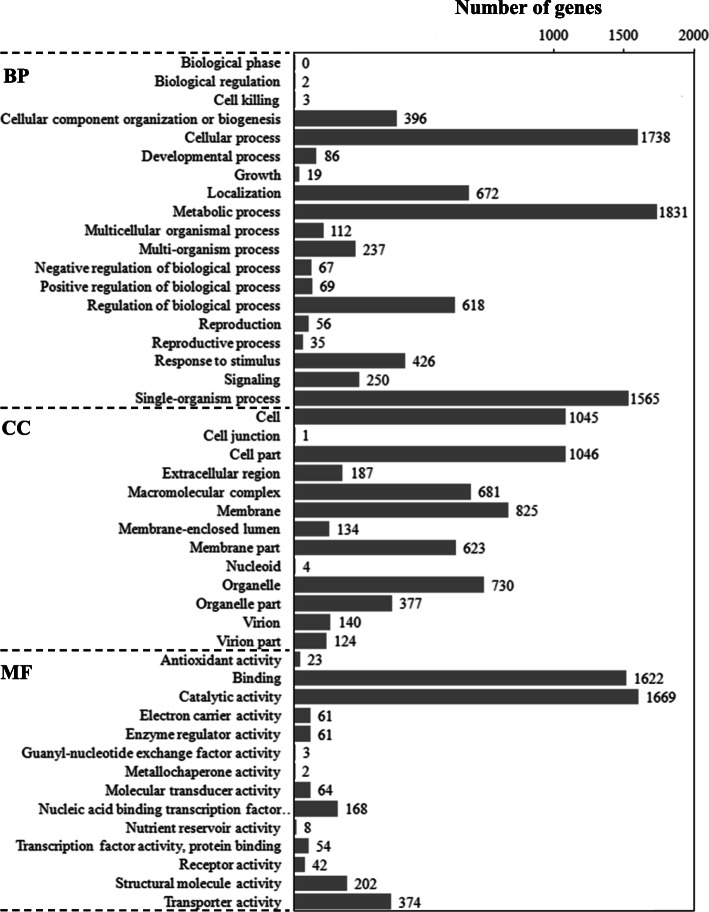
GO functional classification of DEGs. BP: biological process; CC: cellular component; MF: molecular function

**Fig. 7:**
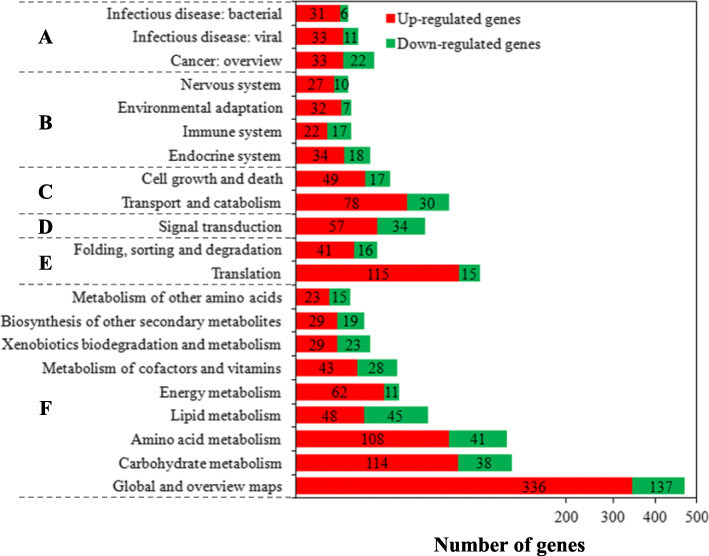
KEGG classifications of DEGs. The top 20 of the second pathway terms are displayed. **a, b, c, d, e** and **f** indicate the first pathway terms. A: human diseases; **b**: organismal systems; **c**: cellular processes; **d**: environmental information processing; **e**: genetic information processing; **f**: metabolism

These DEGs were used for the analysis of gene ontology (GO) and pathway. A total of 2600 DEGs (79.08%) were annotated by gene ontology (GO). GO term analysis for *C. cassiicola* revealed an enrichment of cellular process, localization, metabolic process, regulation of biological process and single-organism process, and binding and catalytic activity (Fig. 6). A total of 891 DEGs (34.27%) were annotated by Kyoto Encyclopaedia of Genes and Genomes (KEGG) pathway analysis, containing 624 up-regulated DEGs and 267 down-regulated DEGs. Gene numbers and IDs for each pathway category are listed in Table S17 in Additional file 2, and the top 20 enriched pathway terms are shown in Fig. 7. The pathway enrichment indicated that the majority of the KEGG-annotated DEGs were involved in metabolism, genetic information processing, cellular processes, organismal systems, human diseases and environmental information processing.

### qRT-PCR validation of selected DEGs

**Fig. 8:**
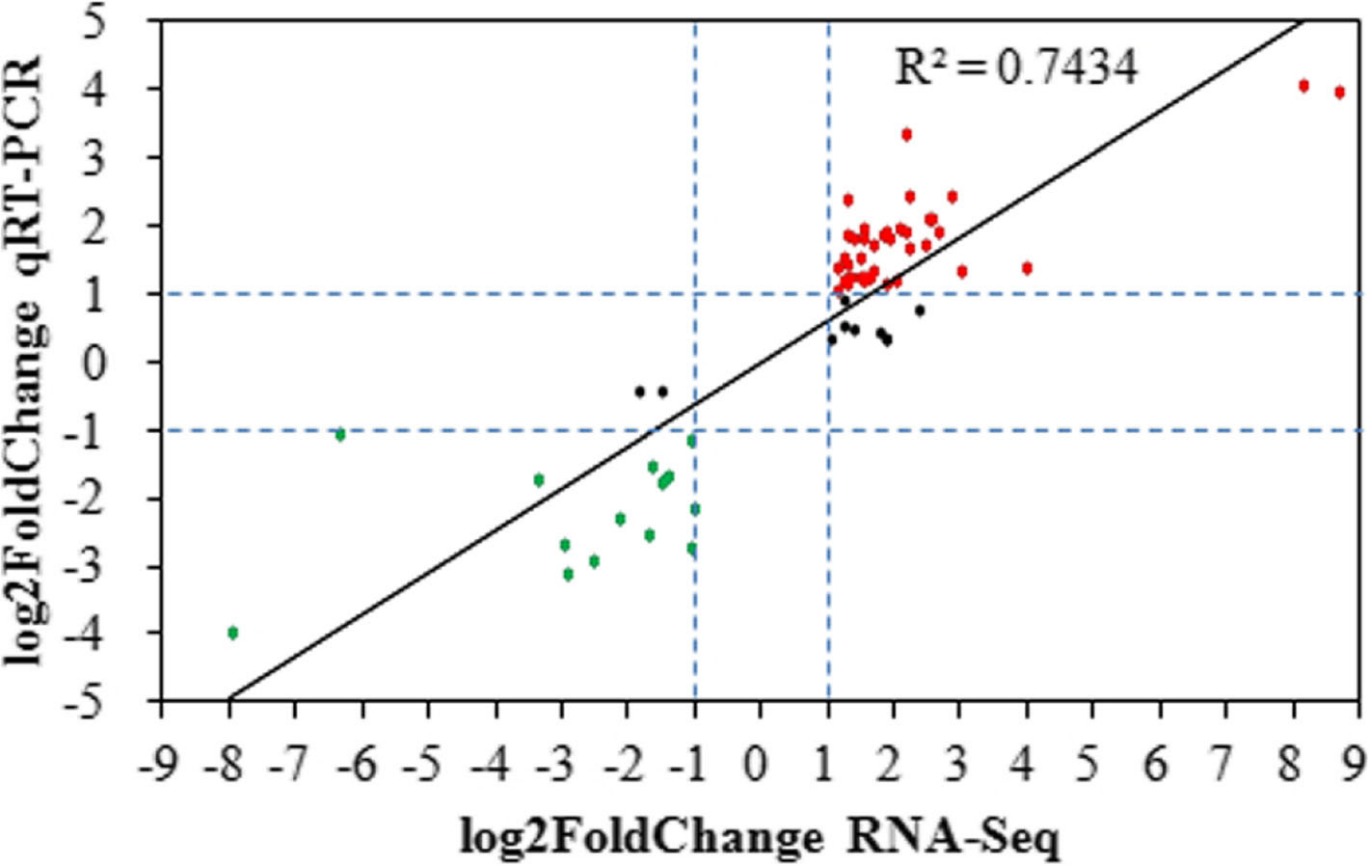
The correlation of RNA-Seq and qRT-PCR analysis for DEGs

qRT-PCR assays were conducted to validate the gene expression patterns of 66 DEGs containing 49 up-regulated and 17 down-regulated genes. As shown in Fig. 8, qRT-PCR data were correlated with the RNA-Seq data (R^2^ = 0.7434). The expression patterns of 56 DEGs were confirmed by qRT-PCR, and 10 were not (Table S18 in Additional file 2). These results showed a high correlation of RNA-Seq and qRT-PCR results, indicating that the RNA-Seq data were very reliable.

## Discussion

*C. cassiicola* is a fungal phytopathogen, capable of infecting hundreds of plants and causing leaf spot disease [1]. In this study, we reported the genome sequence and the spore germination-related transcriptome of a cucumber-sampled *C. cassiicola* isolate. Genome sequencing of the pathogen provided large amounts of important genetic information, which greatly contributed to understanding its evolutionary relationship with other species of phytopathogenic fungi. Additionally, we screened for pathogenicity-associated genes. Many DEGs related to spore germination were identified through RNA-Seq. These results facilitate the study of the pathogenic mechanism of *C. cassiicola* in cucumber.

Phylogenomic analysis showed that *C. cassiicola* had closer genetic affinity with other fungi of the Pleosporales order than of those not of the Pleosporales order fungi, which was similar to previously reported results [24]. Therefore, it was convenient and efficient to identify novel PHI-associated functional factors of *C. cassiicola* and study the pathogenic mechanism of *C. cassiicola* based on comparative genomics with other well researched Pleosporales fungi.

As a phytopathogenic fungus, *C. cassiicola* needs to undergo several crucial and complicated steps to cause host plant disease such as attachment, germination, infection structure formation, invasion and colonization [61]. Fungal pathogens can successfully complete these processes directly via numerous virulence factors, including cutinases, cell wall degrading enzymes, cytomembrane- and cell inclusion-degrading enzymes, mycotoxins, melanin and effectors. The production, transportation and regulation of these virulence factors are mediated by multiple gene pathways, including genes in MAPK, Ca^2+^ and cAMP pathogenic signal pathways, transcription factors, transporters, core genes for the biosynthesis of secondary metabolites, CYPs, etc. [13]. Moreover, there is cross-talk among multiple pathways, which form an extremely complicated regulatory network. Therefore, the high-throughput identification of PHI-associated genes would accelerate the study of the pathogenic mechanism of *C. cassiicola*. As expected, thousands of putative virulence-associated genes in various families or pathways were identified in *C. cassiicola* HGCC, which provided insights into the pathogenic mechanism of *C. cassiicola* in cucumber. Although HGCC, CCP and UM591 were conspecific and close to each other in relationship, as revealed by phylogenomic analysis, there were differences in sequence features of the genomes among HGCC, CCP and UM591 isolates sampled from different hosts. This phenomenon could be explained by the three isolates evolving and developing specific genetic material to adapt to adverse surroundings, which is worth investigating further.

Fungal infection of plants usually requires contact of conidia with the host and subsequent germination [18]. Conidial germination is a genetically programmed and highly coordinated phenomenon that involves the initiation of biochemical activity, an increase in metabolism and the induction of morphological changes [62, 63]. Isotropic growth, also known as swelling, is the first morphological change of conidial germination, which is accompanied by water uptake, cell wall growth, cellular composition changes and a decrease in cytoplasmic microviscosity [64], and by many other metabolic activities. After swelling, chitin in the cell wall becomes polarized, and the fungal cell extends at a restricted area at the tip of the cell, which results in the elongation of the germ tube [65, 66].

Gene expression profiles can provide insight into the molecular and cellular processes of spore germination at either the transcriptional level or at the proteome level. Zhou et al. found that a total of 3026 genes were differentially expressed during spore germination of *Penicillium expansum* using RNA-Seq and iTRAQ, and most of them were involved in metabolism and genetic information processing [18]. Liu et al. identified 66 DEGs during spore germination of *Nosema bombycis* using RNA-Seq [21]. Joise Hander et al. used two-dimensional SDS-PAGE and mass spectrometry to identify a total of 316 spore germination-associated proteins in *Moniliophthora perniciosa*, including the fungal filamentation associated proteins septin and kinesin, a fumagillin-associated transcription factor and polyketide synthase, ATP synthase, binding immunoglobulin protein (Bip), and catalase [67]. Bassi et al. found 1646 DEGs containing toxin-associated genes *nheC*, *cytK* and *hblC* during *Bacillus thuringiensis* spore germination [68]. These research results showed that global transcriptional and protein level analyses were effective tools to understand the molecular and cellular processes of spore germination. Therefore, RNA-Seq was used to explore global gene expression during spore germination of *C. cassiicola* in this study. A total of 3288 genes were found to be differentially expressed, which was similar to the 3026 DEGs observed during spore germination of *P. expansum* [18]. It was found from the GO analysis results of *C. cassiicola* and *P. expansum* that the majority of spore germination-associated DEGs were enriched in cellular processese, localization, metabolic processes, regulation of biological processes, single-organism processes, binding, and catalytic activity. Similar pathway classification results for DEGs were also found between *C. cassiicola* and *P. expansum*, and the majority of KEGG-annotated DEGs were classified in metabolism, genetic information processing, environmental information processing, cellular processes, and human diseases. This phenomenon showed that RNA-Seq data for spore germination were reliable and probably provided valuable information on the molecular mechanism of *C. cassiicola* germination.

Carbohydrate metabolism involves the various biochemical processes responsible for the formation, breakdown and interconversion of carbohydrates, which serve as short-term fuel for organisms [18]. Therefore, it is not surprising that during a relatively short time of germination, 152 DEGs were involved in carbohydrate metabolism, including glycolysis/gluconeogenesis (29 DEGs), the citrate cycle (12 DEGs), the pentose phosphate pathway (15 DEGs), pentose and glucuronate interconversions (16 DEGs), fructose and mannose metabolism (12 DEGs), galactose metabolism (13 DEGs), ascorbate and aldarate metabolism (9 DEGs), starch and sucrose metabolism (17 DEGs), amino sugar and nucleotide sugar metabolism (24 DEGs), pyruvate metabolism (26 DEGs), glyoxylate and dicarboxylate metabolism (21 DEGs), propanoate metabolism (13 DEGs), butanoate metabolism (17 DEGs), C5-branched dibasic acid metabolism (3 DEGs), and inositol phosphate metabolism (5 DEGs). Most of these DEGs were up-regulated, showing that carbohydrate metabolism was essential for *C. cassiicola* spore germination. Similar results were found in the spore germination process of *Penicillium expansum* [18]. Energy metabolism can convert biochemical energy into adenosine triphosphate (ATP). Seventy-three genes were differentially expressed *C. cassiicola* spore germination, and most of these DEGs were up-regulated, which was nearly opposite to Zhou et al.’s results that all DEGs involved in energy metabolism were down-regulated both in the transcriptional and protein levels [18].

## Conclusions

In conclusion, the genome sequence of *C. cassiicola* from cucumber was presented, and thousands of genome-wide virulence-associated genes were mined by homologous searches against multiple functional databases and conserved domain searches. A total of 3288 genes were differentially expressed during the spore germination of *C. cassiicola*. Most of the KEGG annotated DEGs were involved in metabolism, genetic information processing, cellular processes, organismal system, human diseases and environmental information processing. These results not only facilitate the understanding of the molecular pathogenic mechanism of *C. cassiicola* in cucumber and the molecular and cellular processes during the spore germination, but also lay the foundation for disease control.

## Methods

### Fungal strain, hyphae collection, and DNA isolation

*C. cassiicola* (Berk. & M.A. Curtis) strain HGCC, which we isolated from infected cucumber leaves in Shanghai in 2010, was highly virulent to cucumber; thus, it was used for genome sequencing. The isolate was cultured on PDA medium in test tubes at 25 °C for 7 d and then maintained at 4 °C. The HGCC isolate was subcultured on PDA plates at 25 °C in the dark for 10 d, and the mycelium was then collected by peeling it off using a glass slide and ground in liquid nitrogen. Genomic DNA was extracted from finely ground material using the CTAB method [23]. DNA quality control was ensured by 1% agarose gel electrophoresis and an Infinite M200 PRO instrument (Tecan, Switzerland).

### Spore sampling, RNA extraction and sequencing

Spore production tests were conducted on PDA plates. After being activated on PDA plates at 25 °C, the HGCC strain was subcultured on PDA plates at 25 °C for 10 d with 12 h of light and 12 h of dark per day. Five milliliters of sterile ddH_2_O was added to each plate and spores were peeled off using sterile writing brushes and filtered with three layers of sterile gauze to prepare the spore suspension. Spores were collected through centrifugation at 4602×g for 5 min. Spores were resuspended and adjusted to 10^5^ spores/mL with sterile ddH_2_O. The spore suspension was incubated at 25 °C in the dark for 6 h and 12 h to perform the spore germination test. Equal volumes of the germinated spore suspensions incubated for 6 h or 12 h were mixed. The germinated spores in the mixed suspension were collected by centrifugation at 4602×g for 5 min. The germinated spores and ungerminated spores were ground for 1 min using a Geno/Grinder 2010 (SPEX SamplePrep, USA) after being frozen rapidly with liquid nitrogen for the extraction of total RNA. Total RNA was extracted using the TaKaRa MiniBEST universal RNA extraction kit following the manufacturer’s recommended method and stored at − 80 °C. The test was conducted in biological triplicate.

Total RNA was quantified and assessed for purity using a Nanodrop instrument, and the integrity was evaluated using an Agilent 2100 instrument. RNA samples returning an RNA integrity number (RIN) greater than 6.3 were considered acceptable for sequencing. A cDNA library was made from each qualified RNA sample. The libraries were sequenced using an Illumina HiSeq X 10 instrument at the Novogene Bioinformatics Institute, Beijing.

### Whole genome shotgun sequencing and assembly

The whole genome of HGCC was de novo sequenced using an Illumina HiSeq X Ten in 2 × 149 bp paired-end mode with a 400 bp insert sizes of the library at Personalbio (Shanghai, China). High-quality data were obtained from raw data by removing adapter contamination with AdapterRemoval (version 2) [69], collection with SOAPec (version 2.01) [70], and length screening with a threshold of > 50 bp. These high-quality data were de novo assembled using SPAdes v3.9.0 [71]. This whole genome shotgun project was deposited at DDBJ/ENA/GenBank under the accession number RJLO00000000. The version described in this paper is version RJLO01000000.

### Gene prediction, annotation, identification of orthologous gene, and interspecific phylogenomic analysis

Gene structures of *C. cassiicola* genome sequences were predicted using Augustus software with the annotated gene information of *B. cinerea* [72]. Putative secreted proteins were identified by combining Target 1.1 [73], SignalP 4.1 Server [74], TMHMM Server v. 2.0 [75], and Big-GPI software [76]. Putative SCRPs were screened from secreted proteins based on their sequence characteristics such as 20–150 amino acids and at least four cysteines [77]. Protein family classification of *C. cassiicola* was performed by sequence alignment against the Pfam database with profile hidden Markov models (http://xfam.org/) using hmmer 3.1b2 (http://www.hmmer.org/).

Potential virulence-associated genes were identified by local BLASTP searches of *C. cassiicola* HGCC protein sequences against the PHI database (version 4.4, http://www.phi-base.org/) with a cutoff *E*-value of 1e-5. MAPK pathway associated genes of *C. cassiicola* were screened by local BLASTP against *S. cerevisiae* MAPK pathway-associated genes with a cutoff *E*-value of 1e-5 that were confirmed by experiments. GPCRs were identified by local BLASTP against the GPCRDB database (http://gpcrdb.org/) with the best hits and further confirmed by searching seven transmembrane helices with TMHMM Server v. 2.0. Kinases, proteases and transporters were identified by local BLASTP against the KinBase database with a cutoff *E-*value of 1e-10, the MEROPS peptidase database (https://www.ebi.ac.uk/merops/) with a cutoff *E*-value of 1e-20 [78], and the Transporter Classification Database (TCDB) [79] with a cutoff *E*-value of 1e-40, respectively [80]. CYPs and GHs families were classified based on BLASTP alignment against the P450 database (http://drnelson.uthsc.edu/CytochromeP450.html) and CAZy database (http://www.cazy.org/), respectively, with a cutoff *E*-value of 1e-10.

Secondary metabolism backbone genes such as PKS, NRPS and NRPS-PKS were identified in the SMURF system (http://smurf.jcvi.org/index.php) [81]. *C. cassiicola* PKSs and other functionally known PKSs in other fungi were submitted to the SBSPKS database (http://www.nii.ac.in/~pksdb/sbspks/master.html) to modulate and analyze conserved domains. Phylogenetic analysis of PKSs was performed by aligning KS domain sequences and creating a maximum likelihood tree using MEGA 7.0 with Jones-Taylor-Thornton (JTT) model. These functionally known PKS-related toxin biosynthesis pathways included *A. ochraceus* AoLC35–12 (GenBank: AAT92023) for ochratoxin production, *A. alternate* ACTTS3 (GenBank: BAJ14522) for ACT-toxin production, *B. maydis* PKS1 (GenBank: AAB08104) and PKS2 (GenBank: AAR90257) for T-toxin production, *G. zeae* PKS4 (GenBank: ABB90283) for zearalenones production and *G. moniliformis* Fum1p (GenBank: AAD43562) for fumonisin production. Melanin-associated PKSs included *A. fumigatus* Alb1p (GenBank: ACJ13039), *Ceratocystis resinifera* PKS1 (GenBank: AAO60166), *C. lagenarium* PKS1 (GenBank: BAA18956), *C. globosum* PKS-1 (GenBank: AFP82905), *A. rabie* PKS1 (GenBank: ACS74449), *B. maydis* PKS18 (GenBank: AAR90272), *S. turcica* StPKS (GenBank: AEE68981), *B. oryzae* PKS1 (GenBank: BAD22832), and *A. alternate* ALM1 (GenBank: BAK64048).

To compare the gene annotation between *C. cassiicola* HGCC and the other two *C. cassiicola* isolates (UM591 and CCP) or other phytopathogenic fungi, the same pipeline was used for the gene prediction and annotation of the genome for all species. Accession numbers of the genomes are JAQF00000000.1 (GenBanK) for *C. cassiicola* UM591, GCA_003016335.1 (GenBanK) for *C. cassiicola* CCP, GCA_000743335.1 (GenBanK) for *C. lunata*, GCA_000338975.1 (GenBanK) for *B. maydis*, 2,761,201,826 (JGI) for *C. zeae-maydis*, GCA_002267025.1 (GenBanK) for *P. nodorum*, GCA_000359705.1 (GenBanK) for *S. turcica*, GCA_000149985.1 (GenBanK) for *P. tritici-repentis*, GCA_000002495.2 (GenBanK) for *M. oryzae*, GCA_009017415.1 (GenBanK) for *A. flavus*, GCA_000240135.3 (GenBanK) for *F. graminearum*, and GCA_000143535.4 (GenBanK) for *B. cinerea*. To construct an interspecific phylogenetic tree among these fungi, 2831 orthologous protein sequences were screened by local reciprocal Blast search with a cutoff of *E*-value 1e-20. These orthologous proteins were aligned using Clustal W 2.1 [82]. A maximum likelihood tree was created by the concatenated amino acid sequences using MEGA7.0 with the JTT model.

Statistical analysis was conducted by a non-parametric test (Mann-Whitney U test) of SSPS v18.0 using the gene amount of each gene family in *C. cassiicola* (HGCC, CCP and UM591) compared to 10 other phytopathogenic fungi, including *C. lunata*, *B. maydis*, *C. zeae-maydis*, *P. nodorum*, *S. turcica*, *P. tritici-repentis*, *M. oryzae*, *A. flavus*, *F. graminearum*, and *B. cinerea*. The cutoff of significance was set at *P* < 0.05.

### Gene expression in spore germination in *C. cassiicola*

The quality of the raw reads generated from RNA-Seq was checked with FastQC [83]. The clean reads were obtained from the raw reads by removing adapters containing reads, > 10% “N” containing reads, and reads of low quality. The 6 trimmed *C. cassiicola* RNA-Seq libraries were mapped on the predicted CDS of *C. cassiicola* using Bowtie2 with default settings [84]. The number of reads mapped to each gene for each RNA set was calculated from the .sam alignment files derived from Bowtie2. The data of RNA-Seq has been deposited at DDBJ/ENA/GenBank under the SRA accession PRJNA626372.

The counts of RNA-Seq reads over transcripts were used to calculate the fold change of gene expression using DESeq2 [85]. DEGs were selected by the cutoffs on both padj and log2FoldChange. Genes were considered differentially expressed if padj< 0.05 and |log2FoldChange| > 1. Pathway enrichments of DEGs were performed with the KEGG database.

### Quantitative real-time PCR (qRT-PCR) assays

Sixty-six DEGs involved in the cell growth and death of cellular processes were selected for the confirmation of the RNA-Seq data using qRT-PCR (Table S18 in Additional file 2). The *Gapdh* gene was used as the internal control. The first strand of cDNA was synthesized from total RNA of germinated and ungerminated spores using EasyScript All-in-One First-Strand cDNA Synthesis SuperMix for qPCR (One-Step gDNA Removal) (Transgen, China). The qRT-PCR test was conducted with a QuanStudio 6 Flex instrument (Thermo Fisher Scientific, USA). Twenty microliters of reaction mixture contained 2 μL diluted cDNA (1–100 ng), 0.4 μL of each primer (10 μM), 7.2 μL of nuclease-free water and 10 μL of 2 × TransStart® Top Green qPCR SuperMix (Transgen, China). The reaction program was follows: one cycle at 94 °C for 30 s and 40 cycles at 94 °C for 5 s, 60 °C for 15 s and 72 °C for 10 s. The specificities of the amplification products were tested through a melting-curve analysis between 60 °C and 95 °C after each PCR reaction. The relative gene expression data were analyzed using the 2^-ΔΔCt^ method [86]. These qRT-PCR assays were performed with three biological and three technical replicates.

## Supplementary information

**Additional file 1: Table S1** The numbers of genes and PHI-associated genes in *C. cassiicola* and other ascomycetes. Fungal species: HGCC, *C. cassiicola* HGCC; CCP, *C. cassiicola* CCP; UM591, *C. cassiicola* UM591; CL, *C. lunata*; BM, *B. maydis*; CZM, *C. zeae-maydis*; PN, *P. nodorum;* ST, *S. turcica*; PTR, *P. tritici-repentis*; MO, *M. oryzae*; AF, *A. flavus*; FG, *F. graminearum*; *BC, B. cinerea*. ^a^ Average is calculated using the gene amount in the 10 other plant pathogenic fungi (grey column). ^b^*P* value (highlighted in bold for comparisons showing significant differences) is calculated by a non-parametric test (Mann-Whitney U test) of SSPS v18.0 using the gene amount of each family in *C. cassiicola* (HGCC, CCP and UM591) compared to the 10 other plant pathogenic fungi (grey column). The cutoff of significance is set at *P* < 0.05 (highlighted). ^c^ PHI: pathogen-host interaction. ^d^ Percentage: The percentage of PHI-associated gene in protein-coding genes. **Table S2** The number of genes for selected gene families in *C. cassiicola* and other ascomycetes. ^a^ PHI: pathogen-host interaction. ^b^ Average is calculated using the gene/PHI-associated gene amount of each family in the 10 other plant pathogenic fungi (grey column). ^c^*P* value (highlighted in bold for comparisons showing significant differences) is calculated by a non-parametric test (Mann-Whitney U test) of SSPS v18.0 using the gene amount of each family in *C. cassiicola* (HGCC, CCP and UM591) compared to the 10 other plant pathogenic fungi (grey column). The cutoff of significance is set at *P* < 0.05 (highlighted). Fungal species: HGCC, *C. cassiicola* HGCC; CCP, *C. cassiicola* CCP; UM591, *C. cassiicola* UM591; CL, *C. lunata*; BM, *B. maydis*; CZM, *C. zeae-maydis*; PN, *P. nodorum;* ST, *S. turcica*; PTR, *P. tritici-repentis*; MO, *M. oryzae*; AF, *A. flavus*; FG, *F. graminearum*; *BC, B. cinerea*. **Table S3** G protein-coupled receptors in different fungal genomes. ^a^ PHI: pathogen-host interaction. ^b^ Average is calculated using the gene/PHI-associated gene amount of each family in the 10 other plant pathogenic fungi (grey column). ^c^*P* value (highlighted in bold for comparisons showing significant differences) is calculated by a non-parametric test (Mann-Whitney U test) of SSPS v18.0 using the gene amount of each family in *C. cassiicola* (HGCC, CCP and UM591) compared to the 10 other plant pathogenic fungi (grey column). The cutoff of significance is set at *P* < 0.05 (highlighted). Fungal species: HGCC, *C. cassiicola* HGCC; CCP, *C. cassiicola* CCP; UM591, *C. cassiicola* UM591; CL, *C. lunata*; BM, *B. maydis*; CZM, *C. zeae-maydis*; PN, *P. nodorum;* ST, *S. turcica*; PTR, *P. tritici-repentis*; MO, *M. oryzae*; AF, *A. flavus*; FG, *F. graminearum*; BC, *B. cinerea*. **Table S4** Gene-encoding proteins for the MAPK signaling pathway in *C. cassiicola* HGCC. The colour of the protein names indicates the degree of conservation between *S. cerevisiae* and *C. cassiicola* HGCC proteins based on Blastp. Blue, e-value <1e-5 and > 1e-20; Green, e-value <1e-100 and > 1e-20; Red, e-value <1e-100. **Table S5** Gene-encoding proteins for the cAMP signaling pathway in *C. cassiicola* HGCC. **Table S6** Gene-encoding proteins for the Ca^2+^ signaling pathway in *C. cassiicola* HGCC. **Table S7** The number of protein kinases in different fungal genomes. ^a^ PHI: pathogen-host interaction. ^b^ Average is calculated using the gene/PHI-associated gene amount of each family in the 10 other plant pathogenic fungi (grey column). ^c^*P* value (highlighted in bold for comparisons showing significant differences) is calculated by a non-parametric test (Mann-Whitney U test) of SSPS v18.0 using the gene amount of each family in *C. cassiicola* (HGCC, CCP and UM591) compared to the 10 other plant pathogenic fungi (grey column). The cutoff of significance is set at *P* < 0.05 (highlighted). Fungal species: HGCC, *C. cassiicola* HGCC; CCP, *C. cassiicola* CCP; UM591, *C. cassiicola* UM591; CL, *C. lunata*; BM, *B. maydis*; CZM, *C. zeae-maydis*; PN, *P. nodorum;* ST, *S. turcica*; PTR, *P. tritici-repentis*; MO, *M. oryzae*; AF, *A. flavus*; FG, *F. graminearum*; BC, *B. cinerea*. **Table S8** Protease genes classified by the MEROPS family in different fungal genomes. ^a^ PHI: pathogen-host interaction. ^b^ Average is calculated using the gene/PHI-associated gene amount of each family in the 10 other plant pathogenic fungi (grey column). ^c^*P* value (highlighted in bold for comparisons showing significant differences) is calculated by a non-parametric test (Mann-Whitney U test) of SSPS v18.0 using the gene amount of each family in *C. cassiicola* (HGCC, CCP and UM591) compared to the 10 other plant pathogenic fungi (grey column). The cutoff of significance is set at *P* < 0.05 (highlighted). Fungal species: HGCC, *C. cassiicola* HGCC; CCP, *C. cassiicola* CCP; UM591, *C. cassiicola* UM591; CL, *C. lunata*; BM, *B. maydis*; CZM, *C. zeae-maydis*; PN, *P. nodorum;* ST, *S. turcica*; PTR, *P. tritici-repentis*; MO, *M. oryzae*; AF, *A. flavus*; FG, *F. graminearum*; BC, *B. cinerea*. **Table S9** Glycoside hydrolase in *C. cassiicola* and other phytopathogenic ascomycetes. ^a^ PHI: pathogen-host interaction. ^b^ Average is calculated using the gene/PHI-associated gene amount of each family in the 10 other plant pathogenic fungi (grey column). ^c^*P* value (highlighted in bold for comparisons showing significant differences) is calculated by a non-parametric test (Mann-Whitney U test) of SSPS v18.0 using the gene amount of each family in *C. cassiicola* (HGCC, CCP and UM591) compared to the 10 other plant pathogenic fungi (grey column). The cutoff of significance is set at *P* < 0.05 (highlighted). Fungal species: HGCC, *C. cassiicola* HGCC; CCP, *C. cassiicola* CCP; UM591, *C. cassiicola* UM591; CL, *C. lunata*; BM, *B. maydis*; CZM, *C. zeae-maydis*; PN, *P. nodorum;* ST, *S. turcica*; PTR, *P. tritici-repentis*; MO, *M. oryzae*; AF, *A. flavus*; FG, *F. graminearum*; BC, *B. cinerea*. **Table S10** Transporters in *C. cassiicola* and other phytopathogenic ascomycetes. ^a^ PHI: pathogen-host interaction. ^b^ Average is calculated using the gene/PHI-associated gene amount of each family in the 10 other plant pathogenic fungi (grey column). ^c^*P* value (highlighted in bold for comparisons showing significant differences) is calculated by a non-parametric test (Mann-Whitney U test) of SSPS v18.0 using the gene amount of each family in *C. cassiicola* (HGCC, CCP and UM591) compared to the 10 other plant pathogenic fungi (grey column). The cutoff of significance is set at *P* < 0.05 (highlighted). Fungal species: HGCC, *C. cassiicola* HGCC; CCP, *C. cassiicola* CCP; UM591, *C. cassiicola* UM591; CL, *C. lunata*; BM, *B. maydis*; CZM, *C. zeae-maydis*; PN, *P. nodorum;* ST, *S. turcica*; PTR, *P. tritici-repentis*; MO, *M. oryzae*; AF, *A. flavus*; FG, *F. graminearum*; BC, *B. cinerea*. **Table S11** Drug transporters in *C. cassiicola* and other phytopathogenic ascomycetes. ^a^ PHI: pathogen-host interaction. ^b^ Average is calculated using the gene/PHI-associated gene amount of each family in the 10 other plant pathogenic fungi (grey column). ^c^*P* value (highlighted in bold for comparisons showing significant differences) is calculated by a non-parametric test (Mann-Whitney U test) of SSPS v18.0 using the gene amount of each family in *C. cassiicola* (HGCC, CCP and UM591) compared to the 10 other plant pathogenic fungi (grey column). The cutoff of significance is set at *P* < 0.05 (highlighted). Fungal species: HGCC, *C. cassiicola* HGCC; CCP, *C. cassiicola* CCP; UM591, *C. cassiicola* UM591; CL, *C. lunata*; BM, *B. maydis*; CZM, *C. zeae-maydis*; PN, *P. nodorum;* ST, *S. turcica*; PTR, *P. tritici-repentis*; MO, *M. oryzae*; AF, *A. flavus*; FG, *F. graminearum*; BC, *B. cinerea*. **Table S12** Cytochrome P450 genes classified by the CYP family in different fungal genomes. ^a^ PHI: pathogen-host interaction. ^b^ Average is calculated using the gene/PHI-associated gene amount of each family in the 10 other plant pathogenic fungi (grey column). ^c^*P* value (highlighted in bold for comparisons showing significant differences) is calculated by a non-parametric test (Mann-Whitney U test) of SSPS v18.0 using the gene amount of each family in *C. cassiicola* (HGCC, CCP and UM591) compared to the 10 other plant pathogenic fungi (grey column). The cutoff of significance is set at *P* < 0.05 (highlighted). Fungal species: HGCC, *C. cassiicola* HGCC; CCP, *C. cassiicola* CCP; UM591, *C. cassiicola* UM591; CL, *C. lunata*; BM, *B. maydis*; CZM, *C. zeae-maydis*; PN, *P. nodorum;* ST, *S. turcica*; PTR, *P. tritici-repentis*; MO, *M. oryzae*; AF, *A. flavus*; FG, *F. graminearum*; BC, *B. cinerea*. **Table S13** Numbers of backbone genes for the biosynthesis of secondary metabolites in different pathogenic fungi. ^a^ PHI: pathogen-host interaction. ^b^ Average is calculated using the gene/PHI-associated gene amount of each family in the 10 other plant pathogenic fungi (grey column). ^c^*P* value (highlighted in bold for comparisons showing significant differences) is calculated by a non-parametric test (Mann-Whitney U test) of SSPS v18.0 using the gene amount of each family in *C. cassiicola* (HGCC, CCP and UM591) compared to the 10 other plant pathogenic fungi (grey column). The cutoff of significance is set at *P* < 0.05 (highlighted). Fungal species: HGCC, *C. cassiicola* HGCC; CCP, *C. cassiicola* CCP; UM591, *C. cassiicola* UM591; CL, *C. lunata*; BM, *B. maydis*; CZM, *C. zeae-maydis*; PN, *P. nodorum;* ST, *S. turcica*; PTR, *P. tritici-repentis*; MO, *M. oryzae*; AF, *A. flavus*; FG, *F. graminearum*; BC, *B. cinerea*. **Table S14** Putative small, cysteine-rich proteins (SCRPs) in *C. cassiicola* HGCC.

**Additional file 2: Table S15** Sequencing yield from individual libraries per sample. ^a^ CC_0_1, ^b^ CC_0_2 and ^c^ CC_0_3 are triplicate ungerminated spore samples. ^d^ CC_6_12_1, ^e^ CC_6_12_2, and ^f^ CC_6_12_3 are triplicate mixed 6 h- and 12 h-germinated spore samples. **Table S16** 3288 DEGs during spore germination identified by RNA-Seq. ^a^ CC_6_12: mixed 6 h- and 12 h-germinated spore samples; ^b^ CC_0: ungerminated spore samples. **Table S17** KEGG pathway enrichment analysis of DEGs. **Table S18** qRT-PCR for selected DEGs involved in cell growth and death. ^*^ The express pattern of DEGs from RNA-Seq was not validated by qRT-PCR.

**Additional file 3: Fig. S1** Pearson correlation between samples in gene expression levels. CC_0_1, CC_0_2 and CC_0_3 are triplicate ungerminated spore samples. CC_6_12_1, CC_6_12_2, and CC_6_12_3 are triplicate mixed 6 h- and 12 h-germinated spore samples. R^2^ is the square of the Pearson correlation coefficient.

## Data Availability

The data that support the findings of this study are available from [https://www.ncbi.nlm.nih.gov/genome/, Accession No. RJLO00000000] and [https://www.ncbi.nlm.nih.gov/sra/PRJNA626372] but restrictions apply to the availability of these data, which were used under license for the current study, and so are not publicly available. Data are however available from the authors upon reasonable request and with permission of [https://www.ncbi.nlm.nih.gov/genome/, Accession No. RJLO00000000] and [https://www.ncbi.nlm.nih.gov/sra/PRJNA626372]. The dataset(s) supporting the conclusions of this article is (are) included within the article (and its additional file(s)).

## Abbreviations

MAPK: Mitogen-activated protein kinase
GPCR: G-protein-coupled receptor
PHI: Pathogen-host interaction
GH: Glycoside hydrolase
ABC: ATP-binding cassette
MFS: Major facilitator superfamily
DHA: Drug: H+ antiporter
MDR: Multidrug resistance
PDR: Pleiotropic drug resistance
CYP: Cytochrome P450 enzyme
NRPS: Non-ribosomal peptide synthetase
PKS: Polyketone synthase
HK: Histidine kinase
DMAT: Dimethylallyl tryptophan synthase
HYBRID: Hybrid PKS-NRPS enzyme
KS: Ketoacyl CoA synthase
AT: Acyltransferase
DH: Dehydratase
ER: Enoyl reductase
KR: Ketoreductase
SCRP: Small cysteine-rich protein
HR: Hypersensitive response
PAMP: Pathogen-associated molecular pattern
PTI: PAMP-triggered immunity
ETI: Effector-triggered immunity
LysM: Lysin motif
CFEM: Common in fungal extracellular membrane
KEGG: Kyoto Encyclopaedia of Genes and Genomes
DEG: Differentially expressed gene
CDS: Coding sequence
