# Supplementary figures and images for: Genome sequence and spore germination-associated transcriptome analysis of Corynespora cassiicola from cucumber
Source: BMC Microbiol. 2020 Jul 8;20:199. doi: 10.1186/s12866-020-01873-w (PMC7346487; doi:10.1186/s12866-020-01873-w)

**Fig. S1**


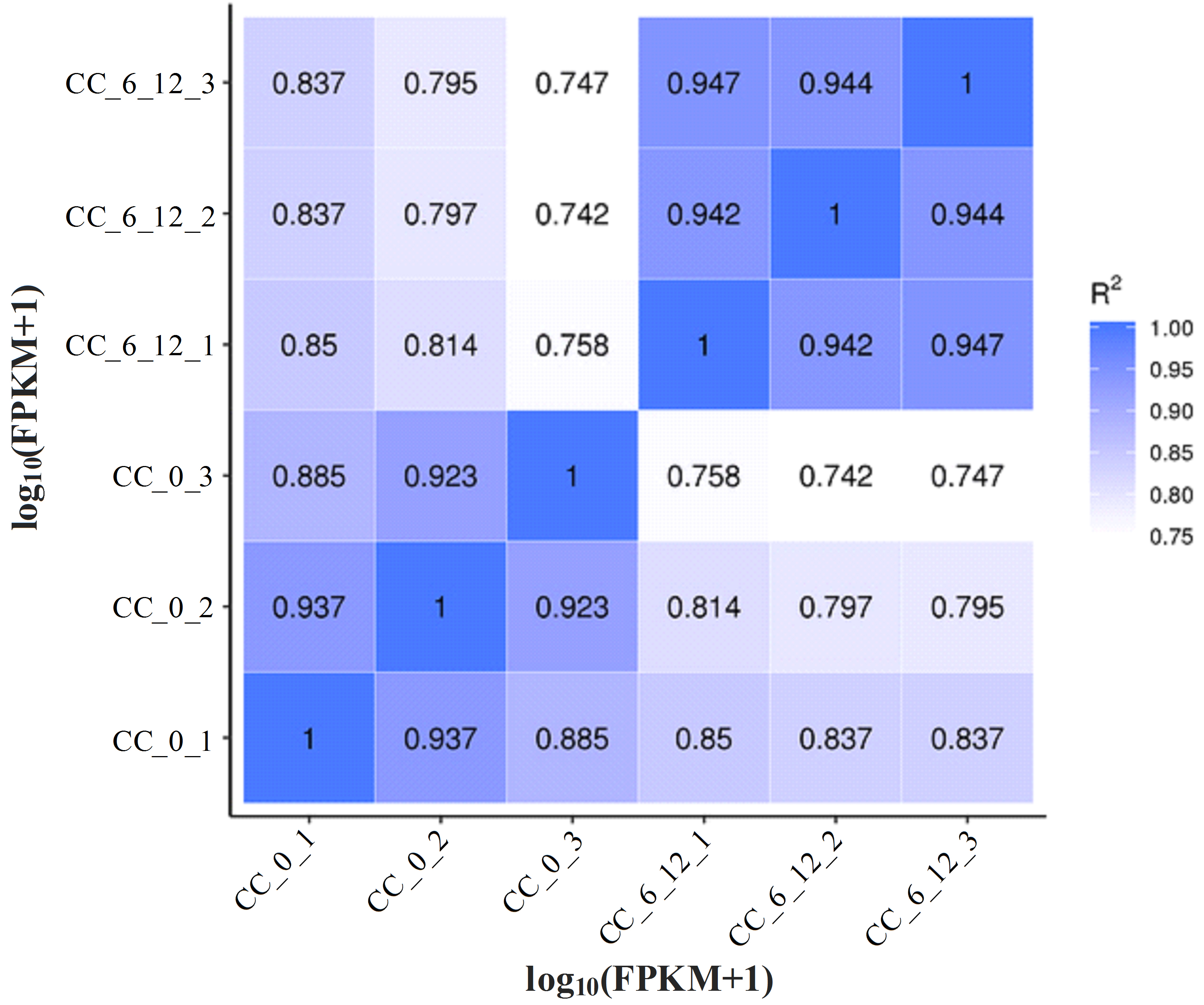

Supplement: Supplementary file 3 — Additional file 3: Fig. S1 Pearson correlation between samples in gene expression levels. CC_0_1, CC_0_2 and CC_0_3 are triplicate ungerminated spore samples. CC_6_12_1, CC_6_12_2, and CC_6_12_3 are triplicate mixed 6 h- and 12 h-germinated spore samples. R2 is the square of the Pearson correlation coefficient. [file 12866_2020_1873_MOESM3_ESM.docx]
